# LncRNA *HOTAIR* in Tumor Microenvironment: What Role?

**DOI:** 10.3390/ijms20092279

**Published:** 2019-05-08

**Authors:** Gerardo Botti, Giosuè Scognamiglio, Gabriella Aquino, Giuseppina Liguori, Monica Cantile

**Affiliations:** 1 Scientific Direction, Istituto Nazionale Tumori, Fondazione G. Pascale, IRCCS, 80131 Naples, Italy; g.botti@istitutotumori.na.it; 2 Pathology Unit, Istituto Nazionale Tumori, Fondazione G. Pascale, IRCCS, 80131 Naples, Italy; g.scognamiglio@istitutotumori.na.it (G.S.); g.aquino@istitutotumori.na.it (G.A.); g.liguori@istitutotumori.na.it (G.L.)

**Keywords:** *HOTAIR*, TME, tumor progression

## Abstract

lncRNAs participate in many cellular processes, including regulation of gene expression at the transcriptional and post-transcriptional levels. In addition, many lncRNAs can contribute to the development of different human diseases including cancer. The tumor microenvironment (TME) plays an important role during tumor growth and metastatic progression, and most of these lncRNAs have a key function in TME intracellular signaling. Among the numerous identified lncRNAs, several experimental evidences have shown the fundamental role of the lncRNA *HOTAIR* in carcinogenesis, also highlighting its use as a circulating biomarker. In this review we described the contribution of *HOTAIR* in the TME modulation, highlighting its relation with cellular and non-cellular components during tumor evolution and progression.

## 1. Introduction

Long non-coding RNAs (lncRNAs) represent a large class of transcribed RNA molecules with a length of more than 200 nucleotides. Like other protein coding genes, they are probably transcribed by RNA polymerase II (RNA pol II), and are polyadenylated [1]. Generally, expression levels of lncRNAs appear to be lower than protein-coding genes [2] and specific to the tissues [3]. Although the function of most of the lncRNAs is unknown, an increasing number of them have been functionally characterized. LncRNAs have been involved in many biological activities such as; (i) the regulation of mRNA processing; (ii) the regulation of transcription, functioning as *cis*- and *trans*-acting modulators of protein-coding gene expression; (iii) the modulation of post-transcriptional control; (iv) the regulation of protein activity; (v) the production of scaffolds for higher-order complexes, such as Polycomb repressive complex 2 (PRC2) [4]. LncRNAs also represent an emerging class of tumor biomarkers being widely documented as to their deregulation in cancer development and progression [5].

Although most of the studies in the literature have shown the role of lncRNAs in tumor cells, a series of recent experimental evidences have suggested investigating their role in tumor microenvironment (TME) [6]. LncRNAs, as well as small noncoding RNAs, are transported in the circulation by extracellular vesicles, mainly exosomes [7]. These play a fundamental role in the interaction between cancer cells and the surrounding environment during tumor progression [8]. In fact, exosomal shuttle RNAs (exRNAs) might be used as a signal to change gene expression patterns in the recipient cell of TME [9,10]. Emerging data suggested that, during tumor disease progression, lncRNAs are involved in tumor-stroma signaling, regulating the phenotype of cancer-associated stromal cells. Stromal lncRNAs are able to induce the release of several chemokines enhancing the metastatic potential of tumor cells [11]. LncRNAs can also influence cancer stem cells (CSC) phenotype and differentiation status within the TME promoting tumor growth, invasion and metastatic progression [12]. In addition, some studies have shown than lncRNAs may be involved both in mechanisms of communication between metastatic cancer cells and extracellular matrix (ECM) and in ECM turnover [13]. 

Many lncRNAs are also expressed in immune cells, and appear to play critical roles in the regulation of immune response [14]. In animal models, lncRNAs are implicated in T cell activation/differentiation [15], and are able to modulate cytotoxicity CD8^+^ T cells recruitment within the TME, promoting metastatic disease. [16]. 

LncRNA *HOTAIR* (HOX Transcript Antisense Intergenic RNA) located on chr.12q13.13 (between *HOXC11* and *HOXC12*) [17] acts as a regulator of chromatin states by binding, with its 5′end, the PRC2. With this mechanism *HOTAIR* can guide PRC2 to target genes related with tumor metastasis in breast cancer [18]. *HOTAIR* further interacts with the methyltransferase specific to histone 3 lysine 27, Enhancer of Zeste homolog 2 (EZH2), and lysine-specific demethylase 1 (LSD1), a central player in epigenetic regulation, through its 5′ end and 3′ end, respectively. Through this mechanism, *HOTAIR* can function as a scaffold directing these proteins to co-occupy the same genomic regions [19]. The same scaffold is able to link E3 ubiquitin ligases with their substrates to accelerate proteolysis [20]. A recent study showed that *HOTAIR* can inhibit Androgen Receptor (AR) ubiquitination, preventing AR protein degradation, and increasing AR protein stability. In fact, *HOTAIR* is able to bind the N-Terminal Domain (NTD) of the AR protein blocking recruitment of the E3 ubiquitin ligase MDM2 that interacts with AR through the same domain [21].

Summing up the experimental evidences, *HOTAIR* is able to: (i) promote the epigenetic activation/repression of gene expression; (ii) affect the target suppression of gene expression through competitive binding to miRNAs; (iii) modify gene expression, at post-transcriptional level, interacting with transcription factors and ribosomes or binding to splicing factors [22]. 

Aberrant *HOTAIR* expression has been detected in several human cancers showing a fundamental role in tumor proliferation, angiogenesis, progression, drug resistance and worse prognosis [23]. In addition, numerous experimental evidences have focused the attention on the potential role of *HOTAIR* as a circulating marker and therapeutic target for cancer patients [24]. 

This review aimed to highlight the potential role of *HOTAIR* in the modulation of TME, focusing attention in particular on its relation with cellular components of TME and ECM proteins. *HOTAIR*, through interaction with microenvironment elements, could play a main role in the activation of specific growth and invasion molecular pathways, and in the regulation of immune response during tumor progression.

## 2. Tumor Microenvironment Cells

The tumor microenvironment consists of different cellular and non-cellular secreted components. Cellular components include cancer-associated fibroblasts (CAF), endothelial cells, immune cells (T-cells, tumor associated macrophages (TAM), dendritic cells, mast cells etc.) and cancer stem cells (CSC) [25]. Secreted non cellular components comprise cytokines, growth factors, metabolites and extracellular matrix (ECM) proteins [26]. All TME elements play fundamental roles in tumor growth and progression [25]. 

### 2.1. HOTAIR and Cancer-Associated Fibroblasts

Fibroblasts, the predominant stromal cell type in the TME, are activated by tumor cells into cancer-associated fibroblasts (CAFs) through the secretion of paracrine growth factors, cytokines, and ECM proteins [26]. CAFs are important mediators of tumor-stroma communication, and are able, directly or indirectly, to promote different biological processes, such as proliferation, invasion, angiogenesis and drug resistance [27]. 

Although the role of lncRNAs in CAF modulation is poorly investigated, some studies suggested that they can contribute to: i) CAFs phenotype and function; ii) induction of epithelial-mesenchymal transition (EMT) in tumor cells, mainly promoting TGF-β signaling [28]. 

A recent study highlighted that breast cancer (BC) cell lines (Estrogen positive (ER+) and Triple Negative (TNBC)), grown in CAFs conditioned media (CAF-CM) lead the production of abundant levels of TGF-B1 cytokine inducing EMT of BC cells. To analyze the epigenetic mechanism by which CAFs induce EMT, several lncRNAs have been measured in BC cell lines revealing the up-regulation of *HOTAIR* in CAF-CM. 

In addition, knock-down of *HOTAIR* in cancer cells is able to induce E-cadherin expression and vimentin and beta-catenin repression, suggesting that CAFs promote EMT and metastasis by activating *HOTAIR* expression. Knockdown of *HOTAIR* in orthotropic mice is able to attenuate the metastatic potential of tumor cells, blocking the crosstalk between CAFs and cancer cells [29]. However, the mechanisms by which CAFs activate *HOTAIR* remain to be addressed. TGF-β is able to assemble a receptor complex that activates Smad proteins (SMAD2, SMAD3 and SMAD4) regulating transcription [30]. In CAF-CM of BC cells, high levels of phosphorylated Smads proteins have been detected. The induced repression of these proteins leads to a 50–60% reduction in *HOTAIR* expression. CAFs could be able to stimulate the direct binding of SMAD2, 3, and 4 to the promoter region of *HOTAIR* inducing its transactivation [29]. In addition, since the activation of CDK5 is essential for CAF-induced EMT, Ren and collaborators also demonstrated that CAFs mediate *HOTAIR* expression and EMT by targeting CDK5 signaling [29]. These observations suggest a novel epigenetic mechanism by which CAFs would modulate EMT and tumor progression by TGF-ß1/CDK5/*HOTAIR* axis.

### 2.2. HOTAIR and Endothelial Cells 

TME endothelial cells are responsible for supporting tumor neovasculature, also participating in several molecular signaling pathways. In fact, tumor-associated endothelial cells display high TGF-beta1 and CD105 expression involved in promoting angiogenesis [31].

Several lncRNAs are described as able to directly or indirectly modulate neo-angiogenesis in TME [32], but between these, *HOTAIR* appears to have a fundamental role in the promotion of angiogenesis during tumor progression. In nasopharyngeal carcinoma (NPC) *HOTAIR* is strongly up-regulated in cell lines and tissues, and its knockdown leads to a significant decrease of several angiogenic factors, such as vascular endothelial growth factor A (VEGFA) and angiopoietin 2 (Ang2) [33]. These data have been also been validated in animal xenograft, suggesting the powerful evidence that silencing of *HOTAIR* can act as an anti-angiogenesis agent to NPC carcinogenesis. *HOTAIR* is directly involved in the promotion of VEGFA transcription. In fact, the results of luciferase assays revealed that HOTAR activated the transcription of 2.3 kb *VEGFA* promoter. Moreover, a functional proteomic profiling identified 14 proteins up-regulated and 29 proteins down-regulated by *HOTAIR* silencing in NPC cells. Between these, Glucose regulated protein 78 (*GRP78*), belonging to the heat shock protein 70 (HSP70) family [34], has been validated as an anti-angiogenetic target of *HOTAIR* in NPC cells [33].

*HOTAIR* is also involved in the angiogenesis modulation of glioma cells. Its downregulation leads to the inhibition of pro-angiogenic activity in in vitro models. Also in this case, the silencing of *HOTAIR* in glioma cells dramatically decreases *VEGFA* expression affecting proliferation, migration and tube formation [35]. Experimental evidences showed that cancer cells are able to secrete extracellular vesicles (EV) to support tumor progression [36]. Numerous lncRNAs, including *HOTAIR*, are found in EVs derived from different cancer cells [37]. Ma and collaborators through RT-PCR analysis confirmed the presence of *HOTAIR* in conditioned medium of glioma cells and experiments using detergents, and RNAse indicated that *HOTAIR* remains stable because it is protected by membranes [35]. These observations suggested that *HOTAIR*, produced by tumor cells, would be able to be released in TME through EVs and transmitted to endothelial cells promoting angiogenesis.

In a non-neoplastic model, a recent study showed that *HOTAIR* is down-regulated in endothelial cells isolated from atherosclerotic plaque in atherosclerosis patients compared with corresponding vascular wall, suggesting that *HOTAIR* plays a protective role for endothelial cells injury [38]. Moreover, serum levels of thymic stromal lymphopoietin (TSLP), belonging to IL-7 like cytokine family, decrease in atherosclerosis patients, and positively correlates with *HOTAIR* expression in endothelial cells. TSLP is able to activate *HOTAIR* transcription through the PI3K/AKT pathway regulating endothelial cells proliferation and migration [38]. 

### 2.3. HOTAIR and Cancer Stem Cells

Cancer stem cells (CSCs) can be a critical component of TME being strongly involved in promoting invasion/metastasis process and drug resistance [39,40]. CSC proliferation can be regulated by many extrinsic factors derived from microenvironment cells. Several studies described that different lncRNAs are able to modulate CSCs proliferation promoting tumor progression [41]. Li et al., showed that *HOTAIR* is able to accelerate liver cancer stem cells proliferation in vitro and *in vivo,* on xenograft mice, through downregulation of the histone methyltransferase SET Domain-Containing Protein 2 (*SETD2*). The *SETD2* gene has been shown to play a tumor suppressor role in human cancer [42]. In fact, cells lacking *SETD2* display microsatellite instability (MSI) and an elevated spontaneous mutation frequency [43]. *HOTAIR* is able to reduce the *SETD2* transcriptional expression through blocking RNApolII catalytic function by dissociating the CREB-P300- RNApolII complex. Therefore, *SETD2* represents a crucial target of *HOTAIR* involved in epigenetic DNA damage repair, abnormal expression of cell cycle-related genes and microsatellite instability (MSI) [44]. 

*HOTAIR* appears as a key modulator of breast CSCs being strongly upregulated in BC CSCs models. *HOTAIR* is able to modulate proliferation, colony formation, migration and self-renewal capacity by negatively regulating miR34a [45]. The searching of the functional region of *HOTAIR* involved in this regulation showed that the full-length *HOTAIR* sequence is required for its regulatory role. Since miR-34a directly targets the stem cell marker *Sox2*, reducing its protein level [46], its mRNA expression has been also evaluated in BC CSCs. *Sox2* appears strongly upregulated in BC CSCs, highlighting its indirect modulation by *HOTAIR*. In conclusion, *HOTAIR* might play a critical role in maintaining CSC self-renewal capacity by negatively regulating *miR-34a* and, consequently, positively *Sox2* [45]. In non-small cell lung cancer (NSLC) patients, high *HOTAIR* expression is associated with drug resistance. In in vitro models, drug resistance induced by elevated *HOTAIR* expression may be caused by the promotion of tumor sphere cell growth and activation of tumor stem cell biomarkers such as *Nanog, Oct3/4, Sox2, c-Myc, β-catenin*, and *Klf4* [47]. In oral squamous cell carcinoma (OSCC), overexpression of *HOTAIR* is tightly associated with the metastatic features of tumor cells, and is able to enhance cancer stemness. On the contrary, silencing of *HOTAIR* strongly attenuates oncogenic and invasion potential in xenograft nude mice. In addition, *HOTAIR* downregulation leads to the decreased expression of mesenchymal-like markers (vimentin, FN1, Snail, Twist and ZEB1) and increased expression of epithelial protein (E-cadherin) in OCSC cells, highlighting its main role of *HOTAIR* in the modulation of EMT features [48].

The role of *HOTAIR* in the modulation of stem-cell phenotypes has been also investigated in normal conditions. Exosomal *HOTAIR* released from gluteal-femoral fat might be able to promote intestinal cells proliferation, inducing both the stem cell and proliferation markers, such as several Wnt pathway-related genes (*Lgr5, Cyclin d1, cMyc*) [49]. *HOTAIR*, together with *miR-10b*, are involved in the malignant transformation of normal liver stem cells. Induced expression of both ncRNAs leads to the loss of E-cadherin and promotes EMT [46]. In bone marrow derived mesenchymal stem cells (MSCs) the overexpression and knockdown of *HOTAIR* interferes with cell differentiation and modulate senescence-associated changes in gene expression and DNA methylation. Targeting of *HOTAIR* to specific sites in the genome seems to be mediated by triple helix formation [50].

### 2.4. HOTAIR and Hypoxic Microenvironment

One of the most investigated features of TME is hypoxia, defined as a reduction in the normal level of tissue oxygen tension [51]. The hypoxic TME is involved in many oncogenic mechanisms, such as decreased DNA repair, increased mutation rate and chromosomal instability [52], cell proliferation signaling [51], invasion and metastasis [53], angiogenesis [54], inflammation and immunity [55] and reduced sensitivity to radiotherapy, chemotherapy and immunotherapy [56]. 

Several experimental evidences highlighted an increased production of exosomes in response to hypoxia [57,58] and oxidative stress [59]. In addition, many studies have demonstrated the aberrant expression of several non-coding RNAs under hypoxic condition. 

Both microRNA and lncRNA in hypoxic microenvironment are able to drive the selection of a stem-like and more aggressive cancer cell population [60,61]. In particular, hypoxia-responsive lncRNAs (HRLs) play crucial roles in regulating hypoxic gene expression by acting as effectors of the indirect response to the transcription factor HIF (hypoxia-inducible factor) or direct modulators of the HIF-transcriptional cascade [62]. 

Different studies describe the role of *HOTAIR* in the hypoxic microenvironment of human cancers. *HOTAIR* is upregulated by hypoxia in NSCLC cells, and it is able to directly interact with HIF-1α through putative hypoxia-responsive elements (HREs) in the promoter region. The binding of *HIF-1α* to the *HOTAIR* promoter in vivo lead the increase of cancer cell proliferation, migration, and invasion [63]. In renal cell carcinoma (Rcc) *HOTAIR* is able to promote tumor progression through inhibition of *miR-217* expression, and modulating the expression of *HIF-1*α and *AXL* receptor tyrosine kinase. In addition, *HOTAIR* knockdown significantly suppresses tumor growth and ki-67 expression, increases *miR-217* levels and decreases the expression of *HIF-1*α and *AXL*, inhibiting tumor growth and EMT [64]. More recent studies showed that *HOTAIR* can be transcriptionally induced upon hypoxic conditions also in other tumor types, including colon cancer, breast cancer, cervical cancer, and neuroblastoma cells [65]. While *HOTAIR* expression is induced upon hypoxia, several *HOTAIR* target genes and tumor suppressors such as *HOXD10, HOXD8, PCDHGA8,* and *PCDHB5* are down-regulated under hypoxic conditions. This mechanism of silencing could be related to the *HOTAIR* recruitment of PRC2 and LSD1 complexes which introduce H3K27-trimethylation and H3K4-demethylation respectively at the above target gene promoters [65]. Through its interaction with *HIF-1a*, *HOTAIR* is also able to induce radio-resistance in cervical cancer cells, suggesting that *HOTAIR*-HIF-1α axis might be a crucial mechanism for radio-resistance modulation hypoxia-induced [66]. 

### 2.5. HOTAIR and Immune Cells

Immune cells represent the most abundant cellular component within TME. In particular, T-cells account for about 50% of intratumoral cells playing a main role in influencing antitumor immunity in both positive and negative ways, depending upon the cell type [67]. 

Tumor-associated macrophages (TAM) cells play a fundamental role in the inhibition of antitumor immunity by secreting suppressive cytokines, leading to an increase of angiogenic process and promoting tumor growth [68]. At same manner, an increased presence of myeloid-derived suppressor cells (MDSC) are strongly associated with tumor progression and poorer outcome [69]. 

LncRNAs play fundamental roles in immune cell development, lineage differentiation and effector function [70]. However, information on the role of *HOTAIR* in immune cells modulation within TME is limited. Hepatoma cell lines with *HOTAIR* overexpression secreted higher CCL2 and this promotes TAM and MDSCs proliferation. CCL2 is required for TAM and MDSCs recruitment in the tumor microenvironment, suggesting that *HOTAIR* has a main role in the modulation of this process promoting tumor growth and metastasis [71]. The analysis of a correlation between CCL2 and *HOTAIR* expression in Gene Expression Omnibus (GEO) data set of HCC, revealed that patients with high expression levels of both markers are associated with advanced clinical stages, suggesting that *HOTAIR* could be a specific target for immunotherapy against CCL2 [67]. Prostate cancer cells treated with androgen deprivation therapy strongly increase the recruitment of mast cells. In turn, infiltrating mast cells are able to decrease AR transcription through *HOTAIR* activation increasing cell invasion via *MMP* expression induction [72]. 

**Figure 1:**
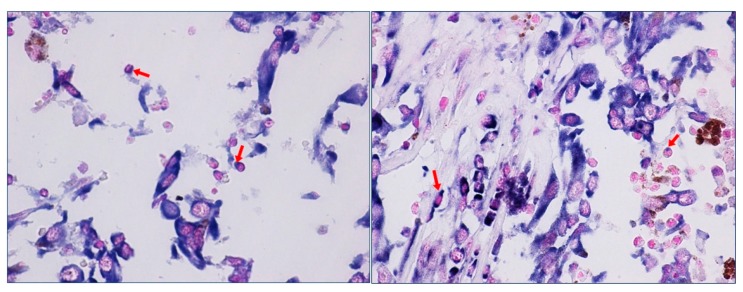
*HOTAIR* expression in immune cells within tumor microenvironment of a melanoma sample. The red arrows indicate membrane projections in which *HOTAIR* staining is predominantly concentrated (magnification 40×).

We have recently highlighted the aberrant expression of *HOTAIR* in metastatic melanoma patients and shown its high expression in lymphocytes surrounding metastatic tumor cells [73]. In our study we clearly showed the in situ expression of *HOTAIR* on immune cells surface within TME. The images of the staining at higher magnification highlight the details of *HOTAIR* staining on the plasma membrane of lymphocytes (Figure 1 (source: ref 74)). *HOTAIR* expression would appear to be associated with specific “vesicle-like” membrane projections. 

However, it is not clear whether the presence of *HOTAIR* on intra-tumor lymphocytes of patients with metastatic melanoma is associated with endogenous production of the lncRNA, or it is the result of a signal transmission between tumor cells and TME cells.

*HOTAIR* expression can be induced in normal immune cells upon treatment with lipopolysaccharide (LPS), an endotoxin able to promote immune and pro-inflammatory response in macrophages. In turn, *HOTAIR* is able to promote several cytokines and inflammatory genes, such as *IL-6, iNOS, TNFα,* and *MIP-1B*, highlighting its critical role in immune response upon LPS-stimulation. *HOTAIR* is also required for LPS-induced degradation of IκBα (nuclear factor of kappa light polypeptide gene enhancer in B-cells inhibitor, alpha) and activation of NF-κB (nuclear factor kappa-light-chain-enhancer of activated B cells) with its nuclear translocation [74]. It is known that NF-κB activation in immune cells enhances inflammation in the TME by increasing the secretion of pro-inflammatory cytokines such as TNF-α and IL-6, and promotes the rapid tumor cells proliferation [75]. 

### 2.6. HOTAIR and Extracellular Matrix

Although extracellular matrix constitutes the non-cellular component of TME, it represents a dynamic and versatile structure able to influence fundamental aspects of tumor cell biology [76]. In fact, during cancer progression, overcoming the physical barriers through the interaction with ECM components is a fundamental step of the invasion/metastatic process. Several studies highlighted an important role of lncRNAs in ECM organization and remodeling [77].

*HOTAIR* has an important function in ECM signaling in breast cancer, above all in invasive “claudin low” molecular subtype. BC cells grown in a 3D culture on a substrate enriched in laminin show a significant up-regulation of *HOTAIR* expression. Not all the different isoforms of *HOTAIR* have been characterized, even if the aberrant activation of the promoter region upstream of *HOTAIR*-N has been mainly implied in invasive breast cancer [78]. Li et al. showed that a novel isoform *HOTAIR*-N is activated in 3D culture of Claudin-low BC cells enriched in laminin. *HOTAIR* expression is induced by increased H3K4me3 and BRD4 binding to the novel *HOTAIR*-N promoter in BC cells attached to ECM, suggesting its main role in ECM organization and tumor invasion pathway signaling [79]. 

The interplay between ECM components and *HOTAIR* has been also demonstrated in lung cancer cells. The enrichment of collagen I in a 3D culture model determines the loss of morphological features of well-differentiated lung adenocarcinoma and the up-regulation of *HOTAIR* expression. On the contrary, the treatment of lung cancer cells with an antibody against collagen I receptor lead to a decrease of *HOTAIR* expression, suggesting that *HOTAIR* expression in tumor tissues is the result of tumor cell response to collagen I, abundantly present in TME [80].

Osteopontin is another component of ECM associated with several biological processes, such as angiogenesis, inflammation, tissues remodeling, but also metastatic progression. In fact, OPN increases pro-MMP2 expression in an NFKb-dependent manner during ECM invasion [81]. OPN is able to induce *HOTAIR* expression in a dose and time-dependent manner in different tumor cell lines, while OPN knockdown decreases *HOTAIR* expression. Activation of the PI3K/AKT pathway seems to be associated with the effect of OPN on the regulation of *HOTAIR*. Moreover, the interferon regulatory factor *IRF1*, a transcriptional regulator of IFN-stimulated genes, might participate in the transcriptional regulation of *HOTAIR*. In fact, promoter activity and ChIP assays confirmed that *IRF1* could bind to the *HOTAIR* promoter and inhibit *HOTAIR* expression [82]. 

## 3. Conclusions

**Figure 2:**
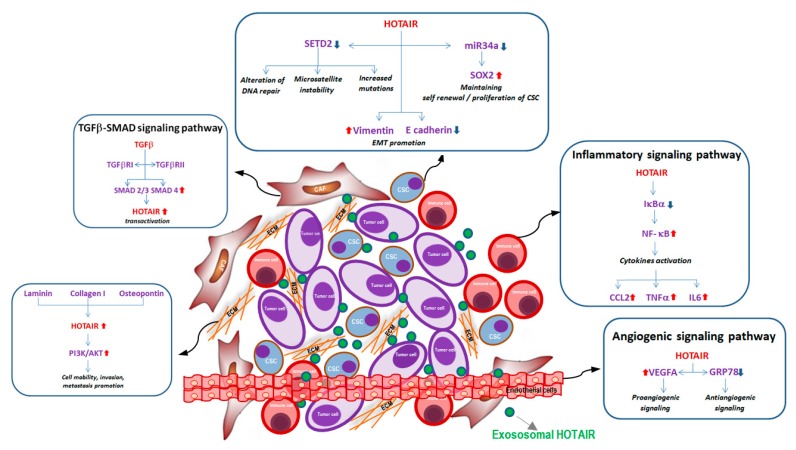
Schematic representation of exosomal *HOTAIR* in tumor microenvironment with details of the main molecular pathways associated with cellular and non-cellular components: (i) In CAF cells: TGF-β assembling the receptor complex (TGFbRI-TGFbRII) activates Smad proteins (SMAD2, SMAD3 and SMAD4) that directly bind *HOTAIR* promoter, inducing its transactivation; (ii) In CSC cells: *HOTAIR* leads to (a) the downregulation of the tumor suppressor gene *SETD2*, promoting microsatellite instability, high mutation rate, and interfering with DNA damage repair, (b) the downregulation of *miR34*a with the consequent induction of stem cell marker *Sox2*; (iii) In endothelial cells: *HOTAIR* induces angiogenesis-promoting *VEGFA* transcription and downregulating the anti-angiogenetic marker *GRP78*; (iv) In immune cells: *HOTAIR* is able to induce the degradation of IκBα with the consequent activation of NF-κB pathways and secretion of pro-inflammatory cytokines; (v) ECM proteins: Laminin, Collagen I, Osteopontin are able to induce *HOTAIR* overexpression modulating PI3K/AKT pathways and promoting cell mobility and invasion.

Although the role of lncRNAs within TME is still poorly investigated, a number of studies suggest the main contribution of *HOTAIR* in TME intracellular signaling and its important contribution in modulating different molecular pathways involved in tumor phenotype modifications during metastatic progression (Figure 2). The evaluation of *HOTAIR* expression in TME could provide fundamental information, not only for the prognosis, but also for the prediction of the therapeutic response of cancer patients.
